# Complete Genome Sequence of a Human Enterovirus 71 Strain Isolated in Wuhan, China, in 2010

**DOI:** 10.1128/genomeA.01112-13

**Published:** 2013-12-26

**Authors:** Zhu Yang, Songya Lu, Jianchun Xian, Jun Ye, Li Xiao, Jun Luo, Ke Zen, Fenyong Liu

**Affiliations:** Institute of Virology, College of Life Sciences, Nanjing University, Nanjing, Jiangsu, Chinaa; Taizhou Institute of Virology, Taizhou, Jiangsu, Chinab; Jiangsu Affynigen Biotechnology, Inc., Taizhou, Jiangsu, Chinac; State Key Laboratory of Virology, College of Life Sciences, Wuhan University, Wuhan, Hubei, Chinad; Department of Liver Disease, People’s Hospital of Taizhou, Taizhou, Jiangsu, Chinae; Institute of Clinical Medicine, People’s Hospital of Taizhou, Taizhou, Jiangsu, Chinaf; School of Public Health, University of California, Berkeley, California, USAg

## Abstract

The complete genome sequence of a human enterovirus 71 strain (EV71/wuhan/3018/2010), which was isolated in Wuhan in 2010, was amplified by a reverse transcription-PCR method and sequenced. Phylogenetic analysis based on the complete genome sequence classified this strain into subgenogroup A.

## GENOME ANNOUNCEMENT

Human enterovirus 71 (EV71), a member of the human enterovirus A species of the family *Picornaviridae*, was first discovered in California in 1969 (1, 2). There has been a significant increase in EV71 epidemic activity throughout the Asia-Pacific region since 1997, and incidents associated with EV71 infections have become serious public health threats (3–5). In healthy adults, EV71 infection normally causes mild illness. However, EV71 has been associated with serious central nervous system infections in infants and young children, leading to aseptic meningitis and encephalitis, with very high mortality observed in China and Southeast Asian countries (6–8). Understanding of the genotypes of the EV71 strains in the epidemic regions is central to the development of novel strategies for the treatment and prevention of the infections and diseases associated with EV71.

In this study, a throat swab sample was obtained from a child in the city of Wuhan in China with a clinical diagnosis of hand, foot, and mouth disease. The presence of EV71 in the sample was confirmed by using a reverse transcription-PCR assay as described previously (9). Furthermore, the virus was isolated by culturing clinical samples in RD cells (ATCC CCL-136). The isolated EV71 strain (called EV71/wuhan/3018/2010) produced typical cytopathic effects (CPE) in RD cells, as observed by the presence of EV71 infection. After propagation and plaque purification in cell culture, six synthetic oligonucleotide primer pairs, which were based on the alignment of available genome sequences of different EV71 strains, were designed to amplify overlapping fragments that span the whole genome of EV71. The sequences of these primers are available upon request. The full-length genome sequence of this virus was established by assembling overlap fragments using the SeqMan program available within the Lasergene 7 package (DNASTAR).

Nucleic acid and protein sequence alignments were carried out with the Clustal W2 program with default settings (10). Phylogenetic trees were generated using the MEGA program (version 5.0) (11), while the bootscan and similarity plot analyses were performed using SimPlot 3.5.1 (12). The genome of the strain EV71/wuhan/3018/2010 was found to contain 7,408 nucleotides (nt), excluding the poly (A) tail. The 5′ untranslated region (UTR) was found to be 743 nt, followed by a single open reading frame (ORF) that encodes a large polyprotein (2,194 amino acids), and the 3′ UTR was 83 nt long. The isolated EV71 strain genome contains 27.62% A, 23.68% C, 23.88% G, and 24.82% U, respectively, with a G+C content of 47.56%. Phylogenetic analyses of the genome sequence, which were carried out by using MEGA5.0, suggest that the isolated EV71 strain belongs to subgenogroup A. Furthermore, strain EV71/wuhan/3018/2010 is more closely related to strain EV71-Hubei-09-China of subgenogroup A (GenBank accession no. GU434678; more than 99.7% nucleotide identity), which was isolated from the same geographic region, than to strains Luan(CHN)-08 (accession no. GQ117124) and EV71/CMU3-1/BJ/CHN/2009 (accession no. JQ410995) of subgenogroup A, which were isolated from different geographic regions in China (13–15).

### Nucleotide sequence accession number.

The full-length genome sequence of the EV71 strain isolated in Wuhan in 2010 has been deposited in GenBank under accession number KF501389.
